# Nortriptyline in knee osteoarthritis (NortIKA Study): study protocol for a randomised controlled trial

**DOI:** 10.1186/s13063-015-0961-1

**Published:** 2015-10-09

**Authors:** Ben Hudson, Jonathan A. Williman, Lisa K. Stamp, John S. Alchin, Gary J. Hooper, Dee Mangin, Bronwyn F. Thompson, Les Toop

**Affiliations:** Department of General Practice, University of Otago, Christchurch, PO Box 4345, Christchurch, 8140 New Zealand; Biostatistics and Computational Biology Unit, University of Otago, Christchurch, PO Box 4345, Christchurch, 8140 New Zealand; Department of Medicine, University of Otago, Christchurch, PO Box 4345, Christchurch, 8140 New Zealand; Pain Management Centre, Canterbury District Health Board, Christchurch, New Zealand; Department of Orthopaedic Surgery and Musculoskeletal Medicine, University of Otago, Christchurch, PO Box 4345, Christchurch, 8140 New Zealand; Canterbury District Health Board, Christchurch, New Zealand

**Keywords:** Osteoarthritis, Analgesia, Nortriptyline, Tricyclic antidepressant, Randomised controlled trial

## Abstract

**Background:**

Osteoarthritis (OA) is a common cause of pain and disability. Currently available analgesics are often insufficiently effective or have unacceptable adverse effects. Tricyclic antidepressants may offer a useful centrally-acting analgesic. Nortriptyline is a readily-available, cheap and comparatively well-tolerated tricyclic antidepressant.

**Methods/Design:**

We will conduct a parallel group, two-arm, participant and investigator-blinded, randomised controlled superiority trial comparing nortriptyline with placebo. Two hundred participants with primary knee OA will be enrolled. Participants will take study medication for 14 weeks. The primary outcome is difference between treatment arms in mean pain score measured on the Western Ontario and McMaster Universities (WOMAC) pain scale at 14 weeks.

**Discussion:**

This protocol describes the first randomised controlled trial of a tricyclic antidepressant in the treatment of OA. The results of the study may have significant implications for the management of this common and painful condition.

**Trial registration:**

The trial was registered with the Australian New Zealand Clinical Trials Registry on 27 June 2014. The trial registration number is: ACTRN12614000683639.

## Background

Osteoarthritis (OA) is the most common joint disease and is a major cause of joint pain and disability [1, 2]. In high-income countries, OA is the fifth leading cause of years lost to disease, [3] and in New Zealand 15 % of 55–64 year-olds and 26 % of 65–74 year-olds have OA [4]. The burden of disease and its associated costs are predicted to rise as the population ages [5]. OA is characterised by pain and loss of function and typically affects the weight-bearing joints of the lower limbs (hip, knee) as well as the hand and spine, but it can affect any joint. OA affecting the knee carries the greatest public health burden [6]. The major pathological features of the disease are degradation of articular cartilage and adjacent bone remodelling. OA is categorised as either primary or secondary: primary OA has no known trigger whereas secondary OA occurs as the result of joint damage from mechanical damage (e.g. fracture), inflammation (e.g. gout and rheumatoid arthritis) or infection. Risk factors for primary OA include advancing age, female sex, and obesity.

OA is predominantly managed in primary care. There is no cure and management is focused on relief of pain and maintenance of function. A range of interventions is available according to the severity of the condition. Initial management consists of patient education, exercise and weight loss, but many patients will also require analgesics [7]. First-line analgesic choices are typically paracetamol and topical non-steroidal anti-inflammatory drugs (NSAIDs); these may be followed by oral NSAIDs or weak opioids [7]. None of these analgesics is ideal: paracetamol, although safe and well-tolerated, may be no more effective than placebo [8, 9]. NSAIDs are effective in reducing pain, [9] but they are nephrotoxic, significantly increase the risk of gastrointestinal ulceration and bleeding, and are associated with cardiovascular events [10]. The long-term use of NSAIDs is, therefore, contraindicated in many patients. Opioids are also used despite poor evidence of their efficacy, and their use is limited by side effects [11]. Ultimately, OA may be treated with joint replacement, but access to this intervention is limited by resource constraints, [12] and patient co-morbidity, and for younger patients delayed joint replacement is desirable due to prostheses’ limited life-spans. Hence, there is a need for more effective and better tolerated pain management for patients with OA.

### Central processing of pain in OA

Pain in OA has historically been attributed to the local nociceptive effects of degeneration of cartilage at the affected joint. Functional imaging has recently revealed areas of the brain that are involved in central processing of pain in OA. Functional magnetic resonance imaging (fMRI) has been used to investigate the central response to painful mechanical stimulation of osteoarthritic knees, and has shown an increase in central activation in brain regions associated with pain [13]. Subsequent treatment of the knee pain with topical lignocaine reduces central activity [13]. fMRI studies of patients with hip OA have also revealed greater central activation amongst those patients with higher levels of neuropathic pain [14]. The central effects of OA pain have also been explored using positron emission tomography (PET) and a similar increase in activity in pain-processing centres in participants with OA pain compared to those without pain has been demonstrated [15].

### Antidepressants as a novel pain treatment in OA

The discovery of central pain-processing activity in OA has suggested a potential new therapeutic approach for pain control in OA [16]. Recent trials of the serotonin and noradrenalin reuptake inhibitors (SNRIs) venlafaxine [17] and duloxetine [18–20] have shown statistically and clinically significant reductions in pain in patients with OA. The study of venlafaxine, however, was very small (*n* = 18) and used a single-blind, placebo run-in design rather than a randomised controlled trial (RCT). The duloxetine studies were larger (*n* = 256 to *n* = 524), were double-blind RCTs, and demonstrated significantly greater reduction in pain and greater improvement in physical functioning with duloxetine than with placebo; however, duloxetine was also associated with significantly more side effects and higher rates of discontinuation than placebo. Furthermore, whilst duloxetine is licensed in New Zealand, it is not subsidised so is not readily available to many of our patients.

Tricyclic antidepressants (TCAs), which are pharmacologically-related to the SNRIs, are commonly used in other chronic pain conditions: for example, in back pain and fibromyalgia, [21–23] but their potential benefits in treating OA pain have not yet been tested in a well-designed RCT. A very small (*n* = 24) cross-over study of imipramine in 1969 suggested a reduction in OA pain [24]. However, this study had several limitations in addition to its small size: the study population was mixed and only 7 participants had OA (other participants had rheumatoid arthritis and ankylosing spondylitis), the treatment periods were short (3 weeks), and there was no washout period between treatment periods. Furthermore, imipramine has a high rate of adverse effects [25]. Nortriptyline is a better-tolerated TCA and is readily available at low cost. TCAs have a range of adverse effects, particularly dry mouth, constipation and drowsiness but nortriptyline is amongst the best tolerated [25, 26]. If nortriptyline proves to have a useful analgesic effect and a tolerable side effect burden it will be a valuable addition to the analgesic options for patients with OA.

### Study aim

To measure the effectiveness, safety and tolerability of nortriptyline compared to placebo in reducing pain and improving function in patients with knee OA.

### Hypotheses

In patients with knee OA, a 14-week course of nortriptyline delivered in addition to usual care will lead to:

1. A significant reduction in OA pain compared to placebo.
2. A significant improvement in physical function greater than with placebo.

## Methods/Design

We propose a parallel group, two-arm, participant and investigator-blinded, randomised controlled superiority trial comparing nortriptyline with placebo.

The study population consists of adult patients living in the Canterbury region of New Zealand with primary knee OA (defined according to American College of Rheumatology (ACR) criteria) causing moderate to severe pain [27]. The ACR clinical criteria will be used as these require clinical findings without x-ray confirmation to confirm the diagnosis of OA. These criteria have slightly lower specificity than the clinical plus x-ray criteria, but their use more closely matches current practice and management guidelines for primary care management of OA. Recruitment will be from the Canterbury District Health Board’s (CDHB’s) orthopaedic service, from primary care, and from public advertisements.

### Inclusion criteria

1. Primary knee OA defined according to ACR classification criteria (knee pain plus 3 of: age > 50 years, morning stiffness lasting < 30 minutes, crepitus, bony tenderness, no palpable warmth) [27].
2. Pain severity of ≥ 20 points on the Western Ontario and McMaster Universities (WOMAC) numerical rating scale (range 0 to 50 points) at the study knee [28].
3. Stable analgesic regime for 2 months before entering the study.

### Exclusion criteria

1. Prior joint replacement surgery on the study knee
2. Intra-articular steroid injection within the previous 3 months
3. Secondary OA (OA due to inflammatory arthritis (e.g. gout, rheumatoid arthritis, juvenile arthritis), septic arthritis or trauma (articular fracture))
4. Known hypersensitivity to nortriptyline or history of adverse reaction to any TCA
5. Current use of nortriptyline or other antidepressants, amiodarone or domperidone
6. Myocardial infarction within 6 months before study entry
7. Heart block
8. Postural hypotension
9. Pregnancy
10. Hyperthyroidism or phaeochromocytoma under current investigation or treatment
11. History of epilepsy or other seizure
12. History of bipolar disorder or manic episode
13. History of increased intra-ocular pressure or history of angle-closure glaucoma
14. Chronic constipation
15. Urinary retention

### Participant recruitment

Potential participants will be identified from the CDHB orthopaedic department and from local general practitioners’ (GPs’) enrolled patient lists. Patients identified from the orthopaedic department will have been assessed as being below threshold for operative treatment for OA. Public advertising will be undertaken in venues typically frequented by older adults: for example, bowling clubs and social clubs. To optimise involvement of Indigenous New Zealanders (Māori), recruitment will be undertaken in *marae* (Māori meeting place) and through the Māori health arm of Ngai Tahu, the principal *iwi* (tribe) in the South Island of New Zealand.

Potential participants identified through these avenues will be sent a letter outlining the study. The letter will include a postage-paid response card which those interested in taking part in the study can return to the research secretary. Individuals indicating willingness to participate will be phoned by the research nurse who will conduct a brief eligibility screen and will invite potential participants to attend a full screening assessment.

### Screening assessment

A screening assessment will be conducted by the research nurse to ensure potential participants meet the inclusion and exclusion criteria. Potential participants’ GPs will be asked to confirm whether there any contraindications to the use of nortriptyline.

The study Clinical Review Team will decide each potential participant’s eligibility for the study. Once eligibility has been confirmed a baseline assessment will be undertaken. Participants will be asked to record NSAID and other analgesic use for 2 weeks before their baseline assessment.

### Baseline assessment, informed consent and assignment of intervention

At the baseline assessment appointment the research nurse will further explain the study, answer any questions and seek informed consent. All participants will provide written informed consent to their participation in the study and will then be assigned a unique sequentially-numbered study identifier according to the order in which he or she is enrolled in the trial. The participant will then complete the baseline assessment and anthropometric measures before the research nurse dispenses the study medication.

### Sequence generation

Participants will be randomly divided into two groups (A and B) of equal size using a computer-generated randomisation schedule with permuted blocks of random size prepared by the study statistician. The randomisation schedule will not be stratified as the risk of important imbalances in prognostic factors is small for a trial of this size [29].

### Allocation concealment and implementation

The study medication (nortriptyline or identical placebo) will be packaged in identical containers. Each container will be pre-labelled (by the study pharmacist contracted to provide the study medication) with a study identifier according to randomisation schedule. The contracted pharmacist will determine which group of participants, A or B, will be allocated to receive nortriptyline. Neither the study statistician nor the contracted pharmacist will have any contact with the participants, nor will they be able to influence treatment allocation. Study numbers and study medication will be issued sequentially to participants.

### Blinding

The study investigators, the research nurse dispensing the medication and assessing outcomes, and the participants will remain blind to the treatment allocation. After the final assessment, the participant’s study arm allocation will be un-blinded and this information will be communicated to the participant’s GP, thus allowing for continued prescription (if desired) of the active medication for those participants in the active treatment arm. The task of un-blinding will be performed independently by a member of the host department staff who is not involved with the study, and allocation will not be revealed to the research nurse or any of the investigators. We will assess the effectiveness of the allocation blinding by asking participants and the research nurse at the final assessment which arm of the study they believe the participant had been allocated to. To prevent accidental un-blinding there will be no contact between the study team and the participant until after data cleaning, database lockdown and analysis are complete.

### Study medication

Nortriptyline dosing has high inter-individual variability: the effective and tolerated daily dose ranges from < 25 mg to > 100 mg [30, 31]. To allow for this, participants will pass through an 8-week blinded dose-adjustment period to allow titration of study medication according to analgesic effect and tolerability. This process closely resembles usual clinical practice when initiating a TCA.

### Dose-adjustment period (weeks 0–8)

All participants will receive capsules containing nortriptyline 25 mg or identical placebo capsules and will be instructed to start study medication at a dose of 1 capsule at night. Every 2 weeks the research nurse will telephone participants and record their response to the study drug: participants will be asked whether they have experienced any change in their knee pain and whether they have experienced adverse effects. Participants who have achieved satisfactory pain relief will be instructed to continue their current dose. If pain relief is inadequate and side effects are tolerated then the participant will be instructed to increase their daily dose by one capsule. If side effects are intolerable then the daily dose will be reduced by one capsule. Dose adjustment will be carried out over 8 weeks to allow participants to reach the potential maximum dose of 4 capsules daily of study medication (a potential maximum dose of 100 mg nortriptyline daily) if required and tolerated. At each of these telephone reviews adverse events will be recorded and serious adverse events (SAEs) will be immediately notified to the Clinical Review Team.

### Steady dose treatment period (weeks 8–14)

Following the initial dose-adjustment period, participants will continue at their maximum tolerated or effective dose for 6 weeks, a period consistent with published recommendations on OA research [32]. During this period participants will not be routinely contacted but they will be encouraged to contact the research nurse by phone if they have questions relating to their study medication.

Participants will be free to use their usual pain relieving medication as prescribed by their GP during the study period. Participants’ GPs will be informed of their patients’ participation in the study and will be asked not to prescribe nortriptyline or any other antidepressants to participants during the study period.

### Outcome measures

In line with recommended practice in OA trials, we will assess outcomes in the domains of pain, physical function and participant global assessment [33].

#### Primary outcome

Difference between treatment arms in mean pain score at 14 weeks, measured using the WOMAC pain subscale and adjusted for pain score at baseline.

#### Secondary outcomes

The following outcomes will be assessed at 14 weeks in a structured interview:

1. Physical function using the WOMAC function subscale
2. Participant-rated global assessment using a visual analogue scale (VAS)
3. Difference in the proportion of participants reporting a treatment effect, defined according to the Osteoarthritis Research Society International (OARSI) set of responder criteria [34]
4. Quality of life using the 36-Item Short-Form Health Survey 36 (SF36) survey
5. Participant-recorded NSAID and other analgesic use in the final 2 weeks of the study period
6. Adverse events will be coded using the Common Terminology Criteria for Adverse Events (CTCAE) [35]. Tricyclic adverse effects will be recorded using the Antidepressant Side-Effect Checklist, [36] measured at 14 weeks.

*The WOMAC knee OA index* measures the three domains of pain, disability and joint stiffness using a set of 24 questions. It is amongst the most widely used measures of lower limb symptoms and has been well-established as a valid, reliable and responsive measure of pain and disability in OA [37, 38]. The WOMAC index used in this study will be version 3.1 in the 11-point numerical rating scale format standardised in English for a New Zealand population. The pain component of the WOMAC index covers five situations: walking on a flat surface, going up or down stairs, night pain, sitting or lying, and standing. The level of pain experienced in each of the 5 situations is scored from 0 to 10 (no pain to extreme pain) to give a combined maximum score of 50. Minimal training is required to administer the WOMAC, and completion time is approximately 12 minutes per participant.

### Baseline assessment measurements

In addition to the primary and secondary outcome measures, the research nurse will also record participants’:

1. Age
2. Sex
3. Ethnicity (using New Zealand Census 2006 categories)
4. Height and weight for calculation of body mass index (BMI)
5. Study knee disease duration
6. Location and number of other osteoarthritic joints
7. Use of assistive devices
8. Current medication use:
  - Analgesics (including NSAIDs)
  - All other medications
9. Other chronic conditions.

### Final assessment

The research nurse will assess each participant at the study clinic at week 14. The following data will be recorded:

1. Pain using the WOMAC pain scale
2. Physical function using the WOMAC function scale
3. Participant-rated global assessment using a VAS
4. Quality of life using the SF36 survey
5. NSAID and other analgesic use in the previous 2 weeks
6. Tricyclic adverse effects using the Antidepressant Side-Effect Checklist
7. Other adverse events
8. Participant’s belief about treatment arm allocation

### Study medication withdrawal

Withdrawal of TCAs (including nortriptyline) may be associated with antidepressant discontinuation symptoms. It is, therefore, recommended that these medications are withdrawn in a gradual fashion rather than stopping abruptly. We will recommend a tapered withdrawal to participants in the active arm of the study at the completion of the study. The daily dose will be reduced by 1 capsule (25 mg nortriptyline) every week. To ensure that the investigators and the research nurse remain blinded, this information will be communicated to the participant in a pre-prepared letter which will be posted to the participant by a non-blinded member of the host department. Participants in the placebo arm will also receive a pre-prepared letter informing them of their treatment allocation and that they can simply stop taking their study medication.

### Sample size

The minimum important clinical difference for a reduction in pain measured using the WOMAC osteoarthritis index has been determined to be about 10 % of the scale maximum, or a total difference of 5 points on the WOMAC pain numerical rating scale (range 0 to 50) [39]. A sample size of 85 per group will give at least 90 % power at a 2-sided significance level of 0.05 to detect a difference in treatment effect of 5 points between the nortriptyline and placebo groups. The sample size was calculated using a pooled standard deviation 10 points estimated from previous studies, [40] and conservatively assuming no correlation between baseline and follow-up scores. This sample size also allows for detection of the minimum important clinical difference in the proportion of participants responding to treatment according to the Outcome Measures in Rheumatology-OARSI (OMERACT-OARSI) criteria [34]. The sample size will be inflated to 100 participants per group (200 in total) to account for a possible 15 % loss-to-follow-up.

### Data management and statistical analysis

Participants’ data will be recorded in individual participant record booklets. Data will be entered into an Access database (Microsoft, Redmond, WA, USA) and then exported to the latest available versions of R and Stata (StataCorp, College Station, TX, USA) [41].

Data analysis will be performed on an intention-to-treat basis with primary and secondary outcomes compared between participants randomised to nortriptyline versus those randomised to placebo regardless of adherence to entry criteria, study medication actually taken, treatment withdrawal, or protocol deviation. A per-protocol analysis will also be performed. Unit of analysis will be at the level of the patient, where patients with OA in both knees will choose the most symptomatic to be the one under study. No interim analyses will be undertaken. All statistical tests will be 2-sided and a level of significance (alpha) of ≤ 0.05 set for all confidence intervals and *p* values. Demographic characteristics and baseline data will be summarised using descriptive statistics, and presented by treatment group. The mean and standard deviation of participants’ WOMAC pain score at 14 weeks follow-up, and change between baseline and 14 weeks, will be calculated for each treatment group.

The primary outcome of the study will be the size of the treatment effect (mean difference in pain between treatment groups at 14 weeks adjusting for differences at baseline), which will be determined using linear regression modelling including treatment group as fixed effect and baseline pain scores as a covariate. A secondary analysis of this primary outcome will be conducted using multivariable linear regression, adjusting for pre-randomisation variables reasonably expected to predict a favourable outcome (duration of disease, medication use at baseline, and use of assistive devices), and participants’ use of other analgesics (paracetamol, NSAIDs, and opioids) in the 2 final weeks of the study period (weeks 12–14). Secondary continuous outcome measures (WOMAC function and stiffness, patient global assessment, and quality of life measures) will be analysed in a similar manner.

As secondary analysis, patients will be dichotomised according to the OMERACT-OARSI set of responder criteria, defined as a high improvement in pain or function, or a moderate improvement in at least two of pain, function, or patient’s global assessment [34]. Incident rates will be calculated for this and other binary outcomes (including use of other pain medications, adverse events, and tricyclic side effects) and compared using chi-square or Fisher exact tests. Generalised linear regression models will be used to calculate absolute and relative differences in risk between treatment groups with 95 % confidence intervals, adjusting for other variables as appropriate. Self-reported treatment dosage over the past 2 weeks will be summarised for each follow-up, as will the number of participants discontinuing treatment and reasons for doing so. The incidence of all suspect serious treatment reactions will be presented in line with the CONSORT 2010 recommendations [42].

Participants who are missing outcome data will be included in the analysis using modern multiple imputation methods. To determine the robustness of results per protocol analysis will be performed, excluding participants with major protocol violations such as cross-over treatments, withdrawals and loss-to-follow-up. Dose-response will be investigated by entering final dose achieved as a predictor in regression models.

### Ethical approval

The study was approved by the Northern A Health and Disability Ethics Committee. Ethics ref 14/NTA/139.

## Discussion

This protocol outlines the design of the first randomised controlled trial of a TCA in the management of OA. As there is currently a paucity of effective and safe analgesics for this common condition, the results of the study may have widespread clinical and public health implications.

## Trial status

The trial was registered with the Australian New Zealand Clinical Trials Registry on 27 June 2014. Trial identifier: ACTRN12614000683639.

Status: recruiting.

## Abbreviations

ACR: American College of Rheumatology
BMI: body mass index
CDHB: Canterbury District Health Board
CTCAE: Common Terminology Criteria for Adverse Events
GP: general practitioner
fMRI: functional magnetic resonance imaging
NSAID: non-steroidal anti-inflammatory drug
OA: osteoarthritis
OMERACT-OARSI: Outcome Measures in Rheumatology-Osteoarthritis Research Society International
PET: positron emission tomography
RCT: randomised controlled trial
SAEs: serious adverse events
SF36: 36-Item Short Form Health Survey
SNRIs: serotonin and noradrenalin reuptake inhibitors
TCA: tricyclic antidepressant
VAS: visual analogue scale
WOMAC: Western Ontario and McMaster Universities
